# The Mechanistic Target of Rapamycin (mTOR) Pathway as a Target of Anti-aging Therapies: The Role of Rapamycin and Its Analogs in the Regulation of Cellular Processes and Their Impact on Longevity

**DOI:** 10.7759/cureus.98514

**Published:** 2025-12-05

**Authors:** Julia Zerdka, Patryk Brasse, Mateusz Piszka, Eliza Kwapien, Maria Kubicka, Jakub Bartkowski, Jan Banach, Hubert Dacyl, Aleksandra Owczarska

**Affiliations:** 1 Medicine, Wojewódzki Szpital Specjalistyczny nr 5 im. św. Barbary, Sosnowiec, POL; 2 Medicine, Samodzielny Publiczny Szpital Kliniczny im. Andrzeja Mielęckiego w Katowicach, Katowice, POL; 3 Medicine, Miejskie Zakłady Opieki Zdrowotnej w Żorach, Żory, POL; 4 Medicine, Szpital Powiatowy w Limanowej Imienia Miłosierdzia Bożego, Limanowa, POL

**Keywords:** anti-aging medicine, healthspan, longevity medicine, mechanistic target of rapamycin, mtor inhibitors

## Abstract

Aging of the body is a complex, multifactorial biological process, leading to a gradual loss of homeostasis, accumulation of molecular damage, and an increase in susceptibility to civilization diseases. In the face of a global aging population, pharmacological strategies are intensively sought that could slow down or partially reverse the aging process. One of the best-understood molecular pathways for regulating lifespan is the mechanistic target of rapamycin (mTOR) pathway, which integrates metabolic, hormonal, and environmental signals. Inhibition of mTOR, through the use of rapamycin and its analogs, consistently prolongs life in numerous animal models, improving age-related physiological functions. Preclinical evidence indicates that rapamycin prolongs the life of animals, improves metabolism, heart function, cognitive abilities, and immunity. In human clinical trials, low doses of rapamycin improve the immune response, reduce markers of skin aging, and are well tolerated. Rapamycin opens a new chapter in research into pharmacological slowing of aging. Understanding its effects on mTOR and autophagy could enable the development of effective interventions to support human longevity and metabolic health in the future, making these substances a promising direction for further research.

## Introduction and background

Modern science is facing numerous challenges of fundamental importance for the future of humanity, starting from the climate crisis, through civilization diseases, to the problem of an aging population. Among these issues, longevity occupies a special place, understood not only as prolonging life, but above all as prolonging the period of biological vitality - healthspan. Aging remains one of the most complex and multifactorial biological processes, involving both the accumulation of molecular damage and the deregulation of homeostatic mechanisms at the cellular and systemic level [1].

Despite enormous progress in knowledge in the field of the biology of aging, the question of the possibility of deliberately slowing it down or even partially reversing it raises great hopes and is a hot topic not only in the scientific community but also in public opinion. Unfortunately, as is often the case with complex problems, the growing interest in the topic of longevity goes hand in hand with the search for simple answers. The emergence of simplistic narratives and the search for the "elixir of longevity" becomes a symbol of this tendency. This review aims to show the complexity of the topic of pharmacological modulation of aging.

In recent decades, a number of molecular pathways that regulate the rate of aging have been identified, including mTOR (mechanistic target of rapamycin) signaling, which is considered a key regulator of cellular growth, metabolism, and energy homeostasis [2]. It is in the context of this pathway that intensive research is being conducted on mTOR inhibitors (such as rapamycin and its derivatives), which in animal models consistently prolong life and improve age-related physiological functions [3-5].

Rapamycin (sirolimus) is an organic chemical compound produced by *Streptomyces hygroscopicus*, which was isolated from soil samples taken on Easter Island Rapa Nui (hence the name of the drug) by Georges Nogrady in the late 1960s [6]. At the molecular level, it acts as a specific inhibitor of the mTOR by forming a complex with the immunophilin FKBP12, which then binds to mTOR and suppresses its kinase activity. As a result, rapamycin downregulates protein synthesis, cell growth, and anabolic metabolism, while promoting autophagy - a cellular process responsible for the degradation and recycling of damaged proteins and organelles. This shift from anabolic to maintenance and repair processes is considered one of the key mechanisms underlying its longevity-promoting effects. Initially, rapamycin was mainly interested in its antifungal properties, then rapamycin was discovered to inhibit the growth of eukaryotic cells, which was used in immunosuppressive and anticancer therapies. A breakthrough discovery in rapamycin research occurred in 2009, when rapamycin was reported to extend the life of mice, which changed the scientific world's perception of aging [7]. Currently, numerous studies are being conducted on the use of the effect of mTOR inhibitors on longevity [8]. Although animal studies are numerous and promising, a significant question arises as to whether a similar effect can be achieved in humans, this topic remains one of the most important and at the same time the most controversial issues in modern experimental gerontology. Therefore, this paper also aims to review and discuss available studies providing evidence for the use of rapamycin in human diseases.

## Review

Methods

This narrative review discusses and summarizes the available scientific literature on the effects of rapamycin on human and animal organisms, covering publications from 2009 to 2025. The literature search was carried out in the PubMed and Google Scholar databases. A combination of keywords was used: rapamycin, mTOR inhibitors, autophagy, longevity, aging, clinical trial, animal study, human study, oxidative stress, neuroprotection, metabolism, and healthspan.

The analysis included 35 publications, including 14 review articles and 21 original studies. Original studies included both animal experiments and studies in human populations, covering different types of projects: randomized controlled and double-blind clinical trials (also using placebo), cohort, prospective, pilot, survey, case reports, and epidemiological reports and ongoing studies. As many as 24 papers were published between 2020 and 2025, with the latest cited study dating back to April 2025. This emphasizes the timeliness of the information cited and indicates the high interest of the scientific community in the subject of prolonging human life.

Inclusion criteria for studies included: publications in English or Polish, studies evaluating the effects of rapamycin on biological, physiological, or health parameters, experimental studies (in animals or humans) and review papers based on primary sources, full-text articles, papers published in peer-reviewed journals or recognized scientific sources. The exclusion criteria included: publications of the nature of comments, letters to the editor, or conference abstracts without complete data, studies with inconclusive methodology or duplicate publications in different databases. The search results have been manually analyzed for compliance with the accepted criteria.

Because this work is a narrative review, no formal risk-of-bias or Preferred Reporting Items for Systematic Reviews and Meta-Analyses (PRISMA) flow diagram was applied. However, care was taken to critically evaluate the quality, relevance, and methodological rigor of the cited studies. 

The role of mTOR in the regulation of cellular senescence

The activity of the mTOR pathway, according to a growing body of genetic and pharmacological evidence, is now recognized as a key factor in regulating the aging process. Inhibition of mTOR has been shown to lead to an increase in life and healthy functioning in laboratory animals [3-5]. The question is: what molecular mechanisms are behind this?

Activation of Autophagy

Autophagy is a key mechanism for degradation of damaged proteins and organelles, essential for maintaining cellular homeostasis. This process is strongly regulated by the mTORC1 complex, which inhibits autophagy initiation by blocking the ULK1 complex under conditions of nutrient abundance. Rapamycin's inhibition of mTORC1 cancels this effect by activating autophagy and supporting amino acid recycling and the removal of damaged structures. With age, autophagy activity decreases, leading to the accumulation of damaged mitochondria and proteins, promoting cellular aging. In many models of organisms (from yeast to mammals), inhibition of mTORC1, among others by rapamycin, has been shown to restore autophagic activity and increase lifespan. Rapamycin stimulates the breakdown of damaged cytoplasmic elements, reduces oxidative stress and supports tissue regeneration, for example in the cardiac tissue. Long-term administration of rapamycin has been shown to increase autophagy, reduce lipofuscin accumulation and improve cardiomyocyte function. In the nervous system, it reduces oxidative stress, apoptosis and accumulation of toxic protein aggregates such as Aβ and phosphorylated tau [6,9,10].

Maintenance of Proteostasis

Protein homeostasis (proteostasis) is responsible for maintaining the proper composition and quality of proteins in the cell. With age, the ability of cells to preserve proteostasis decreases, which promotes the accumulation of damaged proteins and the development of age-related diseases. The mTORC1 pathway, which controls mRNA translation, plays a key role in this process, as its overactivity increases the rate of protein synthesis at the expense of protein quality. Inhibition of mTORC1 by rapamycin reduces translation, improves its accuracy and promotes longer life of organisms, among others by reducing genetic code reading errors and the accumulation of abnormal proteins. This mechanism has been confirmed in the Drosophila and *Caenorhabditis elegans* models. Rapamycin also inhibits cap-dependent translation of mRNA, supporting alternative, stress-resistant translation mechanisms. This increases the production of proteins responsible for stress response and mitochondrial protection (e.g. Hsp70, TFAM), which promotes longevity. In addition, by activating ATF4 and producing hydrogen sulfide (H₂S), rapamycin increases the resistance of cells to oxidative stress. In the context of cancer, its action includes limiting the translation of pro-inflammatory senescence-associated secretory phenotype (SASP) factors (e.g. IL-1, STAT3, NF-κB), which reduces the risk of developing cancer in late life. In conclusion, the inhibition of mTORC1 by rapamycin supports longevity by improving translation quality, reducing toxic protein aggregates, and reducing the pro-inflammatory effects of cellular aging [10-12].

Effects on Mitochondria and Energy Metabolism

Proper mitochondrial activity and homeostasis are crucial for maintaining energy balance and longevity of organisms. Mitochondrial dysfunction is one of the main factors in the development of age-related diseases such as neurodegenerative and cardiovascular diseases. Inhibition of the mTOR pathway by rapamycin has been shown to benefit mitochondrial function through a number of molecular mechanisms. Rapamycin improves the NAD⁺/NADH redox balance, which supports normal oxidative phosphorylation (OXPHOS) and reduces the so-called cellular pseudohypoxia associated with mTOR hyperactivity and HIF-1α accumulation. As a result, lactate production is reduced and the energy efficiency of cells is improved. In addition, inhibition of mTOR promotes mitochondrial biogenesis and increases the expression of key regulators such as peroxisome proliferator-activated receptor gamma coactivator 1-alpha (PGC-1α) and mitochondrial transcription factor A (TFAM), leading to the renewal of mitochondrial populations and improved fatty acid metabolism in senescent tissues, among others in the myocardium and brown adipose tissue [13,14].

Effects on the Immune System and Inflammatory Processes

Aging is associated with weakened immunity and chronic inflammation, which promotes the development of age-related diseases such as cancer, autoimmune diseases, and infections. The mTOR pathway plays a key role in these processes, and its inhibition by rapamycin can restore immune balance. Rapamycin reduces lymphocyte overactivation and production of pro-inflammatory cytokines (m.in. IL-4, IL-17) while increasing the number of regulatory T cells (Tregs). Inhibition of mTORC1 reduces chronic inflammation by reducing the activity of the NF-κB pathway and the NLRP3 inflammasome complex, resulting in a decrease in the production of IL-1β and IL-18 cytokines and a reduction in leukocyte infiltration. Moreover, rapamycin may improve the immune response to infections and vaccinations by increasing CD8⁺ memory lymphocyte count and dendritic cell activity. The immune effect of rapamycin, however, is dose-dependent, lower doses may have immunostimulant, anticancer, antiviral and potentiating vaccine efficacy, while higher doses lead to immunosuppression [10,15].

Preserving Stem Cell Function and Tissue Regeneration

A decrease in stem cell activity is one of the key factors in aging and loss of tissue regenerative capacity. Excessive activation of the mTORC1 pathway leads to impaired proliferation and differentiation of stem cells, promoting their aging and apoptosis. Numerous studies have shown that rapamycin's inhibition of mTORC1 can restore their functionality and improve tissue regeneration. In animal models, rapamycin increased myogenic and chondrogenic differentiation of muscle cells, improved mesenchymal cell function in Klotho-deficient mice. This mechanism is mainly associated with the activation of autophagy, a process that cleanses cells of damaged structures, which increases their survival and resistance to stress. Rapamycin also induces the production of growth factors (IGF-1, VEGF) and reduces inflammation (decrease in IL-1β, TNF-α), supporting cardiac regeneration after infarction and tissue renewal. In conclusion, inhibition of mTORC1 supports the maintenance of stem cell homeostasis by activating autophagy, reducing inflammation and oxidative stress, which may form the basis of therapeutic strategies to delay aging and support tissue regeneration [16,17].

Research on animal models

The first data on the possibility of extending life with pharmacotherapy in mice with heterogeneous genetics date back to 2009 [7]. They gave impetus to the development of numerous more studies proving the effect of mTOR inhibitors on longevity [9]. One experiment investigated the effect on the lifespan of mice with rapamycin and four other drugs: minocycline, β-guanidinepropionic acid, MitoQ and 17-dimethylaminoethylamino-17-demethoxygeldanamycin (17-DMAG), but none of them, except for rapamycin, led to a change in survival [3]. Another study also proved that rapamycin extends the life expectancy of mice and improves their health, as measured by glucose tolerance tests, frailty indicators, gait speed, liver parameters, and weight reduction [4]. The geroprotective effect of rapamycin is due, among other things, to an increase in autophagy, a decrease in age-related intestinal pathologies (reduction of epithelial turnover and prevention of age-related increase in intestinal stem cell (ISC) proliferation, dysplasia and loss of intestinal barrier function), which leads to a longer lifespan in Drosophila [5]. Taken together, these results indicate that inhibition of mTOR by rapamycin opens up prospects for future clinical trials in humans that may allow the development of a pharmacological strategy to slow down aging as a new direction in preventive medicine.

Studies using an Apc Min/+ mouse model corresponding to human familial adenoma polyposis (FAP) have shown that enteral administration of rapamycin (eRapa) effectively inhibits the development of intestinal tumors by inhibiting the mTORC1 pathway and limiting the proliferation of epithelial cells in intestinal crypts. Treatment led to increased survival of the animals, and in some cases the lifespan was equal to that of wild-type mice. Importantly, rapamycin also prevented the development of induced colorectal cancer, confirming its chemopreventive effect. These results indicate that mTOR inhibition may represent a promising therapeutic strategy in the prevention of bowel cancer in individuals with APC mutations and provide the foundation for clinical trials on the use of eRapa in humans [8].

It has also been experimentally shown that rapamycin has potential in the process of tissue regeneration [16,17]. Transplanted stem cells treated with rapamycin may affect the repair of myocardial infarction and the improvement of myocardial function in rats, through rapamycin-activated autophagy, which increases the survival and differentiation of transplanted stem cells in the hostile microenvironment of a heart attack [16]. The results obtained indicate that rapamycin has potential use in regenerative therapies. Future clinical trials should focus on evaluating efficacy in humans, dose optimization, and safety.

One of the challenges of old age is neurodegeneration, unfortunately we are currently helpless in the face of the most common cause of dementia, which is Alzheimer's disease. Rapamycin has been shown to prevent cognitive decline in a mouse model of Alzheimer's disease. The beneficial effects of rapamycin include: reducing β-amyloid deposition, reducing the phosphorylation of pathogenic tau, restoring blood flow in the brain and preserving the integrity of the blood-brain barrier, preventing neuronal loss, and improving cognitive function [18].

Rapamycin also improves cardiovascular function. In a study of 24 middle-aged dogs, echocardiography showed improvements in both diastolic and systolic measures of age-related cardiac function (E/A ratio, fractional shortening, and ejection fraction) in dogs treated with rapamycin [19]. Cardiovascular diseases are a serious problem for the elderly and are the main cause of death, which is why research on the cardioprotective effect of mTOR inhibitors is an interesting direction for the development of science.

The promising results of these experiments in animal models became the basis for the start of clinical trials evaluating the potential of mTOR inhibitors to promote healthy aging in humans.

**Table 1:** Effects of rapamycin in animal models

Reference	Animal model	Drug	End result
Strong et al. (2020) [3]	Genetically diverse UMHET3 mice	Rapamycin, minocycline, β-guanidinopropionic acid, MitoQ, and 17-dimethylaminoethylamino-17-demethoxygeldanamycin (17-DMAG)	Initiation of Rapa at 42 ppm increased survival significantly in both male and female mice. Exposure to Rapa for a three-month period led to significant longevity benefit in male mice only
Shindyapina et al. (2022) [4]	Genetically diverse UMHET3 mice	Rapamycin	Rapamycin extends the median mice life span by 10% and improves their health, as measured by glucose tolerance tests, frailty indicators, gait speed, liver parameters, and weight reduction
Juricic et al. (2022) [5]	Drosophila	Rapamycin	Rapamycin lead to a longer lifespan by increasing autophagy and decreasing age-related intestinal pathologies
Sharp and Strong (2023) [8]	Apc Min/+ mice model	Enteral rapamycin (eRapa)	eRapa inhibits the development of intestinal tumors
Li et al. (2020) [16]	Sprague-Dawley (SD) rats	Rapamycin	Rapamycin-treated transplanted stem cells may enhance myocardial infarction repair and improve cardiac function in rats by activating autophagy, thereby increasing cell survival and differentiation in the ischemic heart environment.
Urfer et al. (2017) [19]	Companion dogs	Rapamycin	Rapamycin-treated dogs showed echocardiographic improvements in both diastolic and systolic measures of age-related cardiac function, including the E/A ratio, fractional shortening, and ejection fraction.

Preliminary human studies

Inhibition of the mTOR pathway prolongs the life of the tested laboratory animals, and in mice it additionally delays the onset of age-related diseases. However, there is a lack of research on whether inhibition of mTOR affects the aging process or its consequences in humans. To date, only indirect mechanisms affecting longevity in humans through immunomodulation have been identified. As we know, the immune system deteriorates functionally with age, this contributes to the aging of the body through reduced removal of aging cells, as well as impaired ability to fight infection, which in turn leads to increased mortality from infectious diseases and cancer [20].

Promising results on the use of rapalogs among the elderly for immunomodulation purposes were brought by two randomized placebo-controlled clinical trials [21,22]. Treatment with everolimus monotherapy or everolimus in combination with RTB10157 for six weeks has been shown to result in a better response to influenza vaccine and fewer infections over the next year compared to the placebo group. As a potential mechanism of the observed effects, an increase in autophagy through inhibition of mTOR along with increased expression of antiviral proteins has been proposed. The observation that immune function may improve after a single six-week period of mTORC1 inhibition has important clinical implications, as it is the elderly who are particularly at risk of dying from influenza [23].

Among the health problems of the elderly population, multifactorial disorders such as sarcopenia, defined as a progressive loss of muscle mass and strength, leading to deterioration of physical performance and an increased risk of falls, are of key importance. This phenomenon is often accompanied by chronic pain and depressed mood or depressive symptoms, which, through mutual interaction, contribute to further limitation of activity and a decrease in the quality of life in old age [24]. The results of the PEARL (Participatory Evaluation of Aging with Rapamycin for Longevity) study seem to confirm hopes for the possibility of pharmacological modulation of the discussed inconveniences related to aging. Significant improvements in lean body mass, reduction of pain symptoms in women, as well as self-assessment of health and emotional well-being in both sexes were observed after administration of rapamycin in low doses, intermittently, for 48 weeks [25]. Similarly, beneficial results of psychiatric treatment were observed with the use of immunosuppression with everolimus without calcineurin inhibitors in heart transplant recipients. Namely, a significant improvement in memory and concentration, mood and quality of life, as well as general psychiatric symptoms [26].

The first evidence that rapamycin treatment can improve the functioning and lower markers of aging in human tissues was provided by a prospective, randomized exploratory study by Chung et al. (2019). It showed that topical application of rapamycin can reduce cellular markers of aging such as the p16^INK4A protein and improve the structural and functional parameters of human skin by increasing collagen VII levels. Additionally, an improvement in the clinical and histological appearance of the skin was noted, confirming the potential anti-aging effect of mTOR inhibitors [27].

**Table 2:** Effects of rapamycin on the human body

Reference	A group of researchers	Drug	End result
Mannick et al. (2014) [21]	218 adults ≥65 years of age	Everolimus (RAD001)	Improving response to influenza vaccination
Mannick et al. (2018) [22]	264 adults aged ≥65 years	Everolimus (RAD001), dactolisib (BEZ235)	Improving response to influenza vaccination
Moel et al. (2025) [25]	114 adults, aged 50-85 years	Rapamycin	Improving lean body mass, self-esteem and emotional well-being, reducing pain symptoms in women
Lang et al. (2009) [26]	9 adult heart transplant recipients, age: 66.1±6.1 years	Everolimus	Improving memory, concentration, mood, and quality of life, as well as general psychiatric symptoms
Chung et al. (2019) [27]	36 adults, age >40 years	Rapamycin	Decreased number of senescent p16^INK4A cells in the skin, increase in collagen VII

Although the results of the studies to date provide promising data on the potential benefits of mTOR inhibitors in humans, their interpretation is limited by the small size of the study population and the short-term follow-up period. Therefore, there is a clear need for large, multicenter randomized trials that will allow for a comprehensive assessment of the long-term effects of mTOR pathway modulation in the context of human longevity and aging.

Side effects, safety, and prospects for anti-aging therapies

Currently, there is a global increase in the representation of older people in the population, so research on the development of geroprotective interventions is essential. Although rapamycin and its analogues have shown efficacy in reversing the effects of aging in preclinical models, multicenter studies are needed, focusing primarily on the safety of therapy in populations particularly burdened with multimorbidity.

To date, the side effects of mTOR inhibitors have been mainly studied among patients taking them as immunosuppressive and anticancer treatments. In these populations, these drugs have many side effects such as: mouth ulcers, delayed wound healing, infections, metabolic and hematological disorders [28,29]. However, these patients are particularly burdened, which is why studies on a healthy population taking small prophylactic doses are necessary.

One survey study of 333 adults using rapamycin outside of medical indications, mainly for longevity effects, and 172 people in the control group looked at potential side effects in this group. Most subjects used rapamycin at a dose of 6 mg once a week. The use of the drug was generally well tolerated, the only significantly more common side effect in the group of rapamycin users was mouth ulcers. There was also a tendency for more frequent infections (respiratory, skin, fungal, and urinary), but it did not reach statistical significance. On the other hand, a few ailments (abdominal pain, muscle tension, eye pain, anxiety, depression) occurred less frequently than in the control group. However, this is a survey study, which is a limitation in its interpretation [30].

Long-term use of rapamycin at low doses seems to have a good safety profile, and the most dangerous side effect may be mouth ulcers. Adverse events in rapalog studies were generally classified as mild or moderate and were reversible upon discontinuation of treatment [31]. Even an unsuccessful suicide attempt (taking 103 mg of sirolimus) did not cause any serious consequences other than a mild change in total cholesterol [32].

The prospects for impacting longevity in humans using mTOR inhibitors are currently being extensively studied. Contrary to numerous preclinical data, clinical trials in the human population are only beginning to provide preliminary evidence regarding safety, efficacy, and possible endpoints for aging.

One of the projects is the ERAP (Evaluating Rapamycin Treatment in Alzheimer’s Disease Using Positron Emission Tomography) study - evaluation of rapamycin treatment in Alzheimer's disease using positron emission tomography, which is a phase IIa trial using imaging biomarkers to assess the effect of rapamycin on glucose metabolism in the brain in people with early Alzheimer's disease. This study may provide the first clinical evidence for the neuroprotective potential of mTOR inhibition in the context of neurodegeneration and central nervous system aging and potential treatments modifying the course of Alzheimer's disease. The study will also assess changes in a number of age-related pathological processes, such as loss of bone mineral density, retinal degeneration, atherosclerosis, and heart disease [33].

In parallel, a randomized, placebo-controlled study is being conducted on the effect of intermittent sirolimus (6 mg/week) on muscle strength and endurance in elderly individuals following a training program. In addition, biochemical and hematological parameters will be monitored, including complete blood count, urea and electrolyte concentrations, liver tests, glycated hemoglobin (HbA1c), lipids, serum insulin-like growth factor 1 (IGF-1) and high-sensitivity C-reactive protein (hs-CRP) concentrations. This project represents an important step towards understanding the synergies between pharmacological modulation of the mTOR pathway and physical activity and their potential importance in the prevention of sarcopenia and the improvement of motor functions in the elderly [34].

Meanwhile, an ongoing pilot study on the effects of rapamycin on ovarian function and prolongation of reproductive life in women opens up a new avenue for research on reproductive aging. If the results from animal models are confirmed in humans, interventions targeting mTOR have the potential to modify the rate of reproductive aging and delay the onset of menopause. This would have far-reaching health and social implications, as the postmenopausal period is associated with a higher risk of osteoporosis, cardiovascular disease, and cognitive decline [35].

Together, these studies mark a new stage in the clinical evaluation of the geroprotective effects of mTOR inhibitors. Although they are small in scale and involve limited populations, they provide an important foundation for future multicenter, hard-endpoint studies aimed at determining the safety, efficacy, and optimal dosing strategies of these interventions in the context of healthy human aging.

## Conclusions

Rapamycin is one of the most intensively studied compounds with potential effects on longevity. Available experimental data indicate that inhibition of the mTOR pathway and activation of autophagy lead to improved cellular homeostasis, reduced oxidative stress, and a slowing of aging processes across multiple model organisms.

Current clinical studies in humans, although limited in number and involving small populations, suggest that low doses of rapamycin may enhance immune function, reduce visible signs of skin aging, and positively influence well-being and metabolic parameters.

Despite these promising findings, knowledge regarding the long-term safety, efficacy, and optimal dosing regimens of rapamycin remains limited. Further, multicenter, randomized clinical trials are needed to determine whether modulation of the mTOR pathway can represent an effective and safe strategy to support healthy human aging.
